# Annotations

**Published:** 1898-02-19

**Authors:** 


### Annotations.

### Exemptions from the Duty of Notifying Infectious Disease.

The evidence given before the Government inquiry concerning the outbreak of typhoid fever at Maidstone has given publicity to an absurd exemption to the operation of the Infectious Diseases Notification Act, which goes far to diminish its efficiency as a means of tracing out the diseases which it was framed to deal with. According to the evidence of the medical officer of health he had not received any notifications from the barracks or the gaol or the post office, as establishments belonging to Her Majesty are exempted from the Act! How far the ægis of the post office would protect an infectious postman in distributing small-pox along with his letters we should not like to say. We fancy the section of the Public Health Act dealing with "exposure" would quickly lay him by by the heels; but it is a matter of some concern that the sanitary authority should have no means of ascertaining the presence of infectious disease either in barracks or in gaols. So far as relates to the spread of infection this is especially the case in regard to gaols, from which prisoners must be discharged on the expiry of their sentence; but in the tracing of an epidemic, such, for example, as that of milk typhoid, which recently occurred at Clifton, it might be a matter of the most serious moment that whole groups of persons resident in the district are exempted from the provisions of the Act, and that the sanitary authority should thus be kept in the dark in regard to the presence or otherwise of the disease amongst them.

### Steaming Instead of Cremation.

Cremation is no doubt a wasteful process. The substances cremated are got rid of and any injurious material is destroyed, and thus, in a sanitary sense, the public gets its return; but economically it is an absolute loss, the commercial value of the fuel used and the manurial value of the substances cremated being entirely thrown away. When used as a substitute for burial in the disposal of the dead this, perhaps, does not matter very much; but there is much cremation which has no concern with human bodies. Slaughter-houses and fish-markets produce large quantities of refuse, the disposal of which is a great difficulty in town sanitation. The same is the case in regard to many business processes connected with the preparation of food stuffs; and if we are to look forward to a time when the inspection of meat and other kinds of food shall be carried out with greater vigour and intelligence than is the case at present, we must also look forward to a time when large quantities of condemned food will have to be got rid of somehow. To bury these things is ineffectual, and even dangerous; to burn them is wasteful, besides being by no means easy of accomplishment without causing a nuisance in the process. To destroy infection and to deprive these diseased and decomposing bodies of all power of doing harm the glowing heat of a furnace is, however, by no means necessary. A steam heat does all that is required. So at Essen, in Westphalia, instead of cremating all this refuse, they just steam it, extracting the gelatine and the fat, and using the residue as a harmless and well-cooked manure. Nay, it is even said that the pigs (poor piggies!) will eat it; so it all comes in again. The process is simple enough. There is a jacketed, steam-heated cylinder, in which a perforated cylinder revolves. Into the inner cylinder the refuse is thrown, steam is turned into the outer jacket, and thus the whole is warmed, the vapours which are given off being pumped out and burned in a furnace. Then steam is turned into the cylinder where the revolving drum is grinding up and centrifugalising the refuse. This is thus thoroughly heated, the fat and gelatine flow away, and ultimately, the steam being turned off from the inner cylinder, air is passed through it, by which the well-cooked compound is dried, and any disagreeable exhalations are carried into the furnace for consumption. Thus all is well, and everything is saved. The evolved gases help the furnace; the gelatine can be used for size, perhaps even may be made up into little packets for the making of "calves' foot jelly"; the fat can be put to various purposes—shall we suggest margarine as a possible destination?—while the residue is sold to the farmer to be turned by him into beans or bacon, according to his taste. Nottingham is, we understand, about to adopt this system of steaming, instead of cremating, the refuse of its abattoirs.

### The Right to Practise.

Many of us have thought that the benefit which, as medical men, we derive from being registered is restricted to the fact that we can recover fees, can hold appointments, can sign certificates, and are exempt from serving upon juries. Mr. Victor Horsley, however, has much more extended views as to the rights given by entry on the register. He maintains that not only is the right to practise medicine, surgery, and midwifery a privilege conferred under the Medical Acts by registration, but that "no one but a registered person possesses such a right." He arrives at this conclusion by virtue of the legal maxim that "if anything is expressly asserted the converse is thereby excluded;" for does not the Medical Act of 1886, in its sixth section, expressly say, "On and after the appointed day a registered medical practitioner shall, save as in this Act mentioned, be entitled to practise medicine surgery, and midwifery in the United Kingdom." Hence, according to Mr. Victor Horsley, the converse is excluded with equal preciseness, and "a person who is not on the register does not possess the right to practise." All this seems deliciously simple, and we can but express our astonishment at the British Medical Association keeping a standing committee constantly sitting (excuse the bull) on the perennial question of the Amendment of the Medical Acts if facts are as Mr. Horsley supposes; for what more can we expect any Parliament to give us, and, in fact, what more can we want, than an exclusive right to practise medicine, surgery, and midwifery throughout the United Kingdom? Why trouble about Midwives' Bills or any other pettifogging reforms if we really have the ball at our feet in this way? Clearly there is only one little detail required to make us all happy and contented (as members of such a close corporation surely ought to be), namely, that Mr. Horsley should persuade the judges that his reading of the law is the correct one.

